# Extra-ocular retinoblastoma: about 12 cases followed at the Mohamed VI university hospital of Marrakech

**DOI:** 10.11604/pamj.2016.25.131.8599

**Published:** 2016-11-02

**Authors:** Soltani Leila, Hajji Ibtissam, Essafi Hafsa, Moutaouakil Abdeljalil

**Affiliations:** 1 Ophthalmology Department, Mohamed VI University Hospital, Marrakech, Morroco

**Keywords:** Retinoblastoma, extra-ocular, proptosis, orbital cellulitis, extrascleral involvement, enucleation

## Abstract

Retinoblastoma is the most frequent childhood intraocular tumor. The aim of our study is to evaluate the clinical features and management of extra-ocular retinoblastoma in the Mohamed VI university hospital of Marrakech. Retrospective case series, the patient's records were reviewed for patient and tumor features, ocular management, histopathological findings, and patient survival. Over a period of three years, 35 eyes were diagnosed with retinoblastoma; 12 children (16 eyes) (46%) had extra-ocular retinoblastoma. Mean age was 27 months, 60% were males. Six cases had unilateral tumor, five bilateral and one case of trilateral retinoblastoma. There was no positive family history, proptosis was the mean mode of presentation (41,6%) followed by staphyloma (25%) orbital cellulitis (25%) and hyphema(8,3%). The median lag period was 18 months. On imaging and histopathological analysis, there was extrascleral involvement in 41.6%, involvement of orbital part of optic nerve (75%), of orbital muscles (50%) and eyelids in 16.6%. the surgical treatment included according to the degree of extension enucleation (75%) or exenteration (25%) associated to chemotherapy in all cases and one case of external beam radiation. There were 2 cases of orbital recurrence, one death and no metastases at 30 months follow-up.Orbital retinoblastoma still stands as a tall challenge requiring multi-modal and multi-disciplinary approach. Although the survival has increased over the last few years, lack of access to medical facilities, lack of education about the need for early medical attention and cultural resistance to enucleation continue to contribute to an epidemic of extra ocular disease at diagnosis in the developing world.

## Introduction

Retinoblastoma is the most frequent childhood intraocular tumor with an approximately incidence of 1/15000-20000 birth in the world [1]. In north Africa, it is the most important ocular malignancy, It is bilateral in about 25-35% of cases [2]. The recent advances for early detection and treatment like identification of genetic mutations, use of chemoreduction to minimize the size of the tumor, identification of histopathologic high risk features following enucleation and provision of adjuvant therapy to reduce the incidence of systemic metastasis, and aggressive multimodal therapy in the management of orbital retinoblastoma have contributed to improve outcomes in terms of better survival [3]. In neglected or untreated cases retinoblastoma can demonstrate extra-ocular spread primarily through the optic nerve and also through the sclera [4]. Orbital retinoblastoma is one of the major contributors to mortality and carries a poor prognosis for life [5, 6]. Though it is a rare clinical presentation in developed countries ranging from 6.3% to 7.6% [7], it is not an unusual feature in developing and under developed world [8], mortality linked to orbital retinoblastoma in these countries is still high owing to late presentations compounded by socio-economic factors with the mortality reported as high as 50-90% [9, 10]. Previous studies in morocco had reported epidemiological and clinical characteristics of retinoblastoma [2, 3]. The aim of our study is to present data over three years about clinical presentation, therapeutic care and outcomes of children presenting orbital retinoblastoma treated at the Mohamed VI university hospital of Marrakech, which is a referral and tertiary care center in the south of morocco, to understand various reasons for the delayed presentation and to compare our results with those reported in the literature.

## Methods

A retrospective case series of all children seen at the eye unit and the pediatric oncology unit of the university hospital Mohamed VI of Marrakech with the diagnosis of orbital retinoblastoma from November 2011 to June 2015 was determined from clinical records of the hospital. Data was compiled on epidemiological characteristics, clinical examination, investigations, histopathology findings, treatment given and their outcomes. The information collected included demographic features, laterality, family history of retinoblastoma, the initial sign noticed, the duration of lag period (the time between the onset of symptoms and diagnosis), any consultation taken in this period and the reason for the delayed presentation. Clinical examination was performed with complete ocular examination including dilated ophtalmoscopy with scleral indentation under general anesthesia. The posterior segment findings included tumor size and location, number of quadrants involved with tumor and extent of retinal detachment. The detail of imaging studies including eye ultrasonography, computed tomography (CT) and magnetic resonance imaging (MRI) of the orbit and brain were recorded. The recorded details included tumor thickness, intra tumoral calcification and evidence of optic nerve, sclera and orbital invasion by the tumor. Ultrasound abdomen and X-ray chest view was done for metastasis analysis for all patients. The Reese Ellsworth classification and international classification of retinoblastoma were used for staging the tumors. The histopathological variables were documented: endophytic and exophytic growth, the size of the tumor, tumor necrosis, the degree of calcification, tumor differentiation, and the presence or absence of fibrosis. The extent of a tumor was established by assessing the involvement of different intraocular structures. Choroidal extension was expressed in progressive grades, optic nerve involvement was expressed in terms of tumor cell invasion of either prelaminar, laminar, or postlaminar optic nerve and involvement of the cut surgical end the optic nerve. We included in cases of extra-ocular retinoblastoma: patients presenting clinical or radiological detected orbital extension of an intraocular tumor, choroidal or extrascleral involvement, invasion of the optic nerve on histopathological evaluation of enuceated eyes and cases of orbital recurrence. Treatment given included surgery by enucleation or exenteration (removal of the entire contents of the orbit) or combined surgery chemotherapy and external beam radiation. Patients with suspected clinical and/or radiological extraocular retinoblastoma received neoadjuvant chemotherapy, defined as chemotherapy administered before enucleation to reduce tumor size. The patients who did not receive any treatment before enucleation were those who had only a histopathological involvement of the choroid or the optic nerve detected after surgery. The chemotherapy used included the recommended protocol of VEC (Vincristine, Etopside, Carboplatin) or VC (vincristine+ciclophosphamide), external beam radiation was performed in case of orbital recurrence. All patients were monitored for the control of the contra lateral eye and for orbital recurrence and metastatic disease by clinical examination, brain and orbit MRI every 3-6 months. Statistical analysis was performed using the statistics software SPSS for windows. Data are represented as means/SD when the distribution was normal and median with range when the distribution was not normal.

## Results

**Figure 1:**
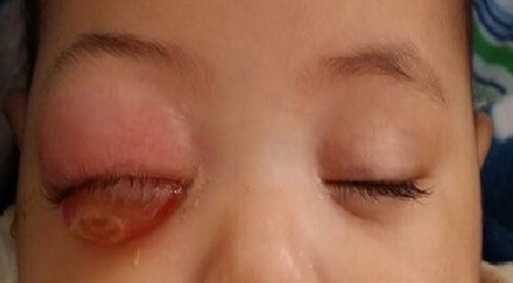
Clinical appearance of orbital cellulitis of the right eye revealing an orbital retinoblastoma

**Figure 2:**
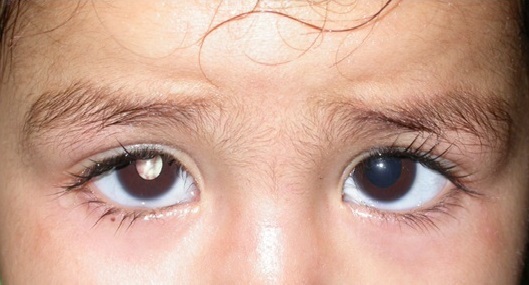
Leucocoria as a mode of presentation of an Unilateral retinoblastoma of the right eye

**Figure 3:**
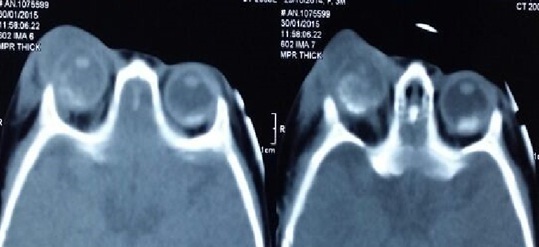
CT scan axial cuts of a bilateral orbital mass, optic nerve extension and orbital cellulitis of the right eye revealing a bilateral retinoblastoma with extra-ocular involvement

**Figure 4:**
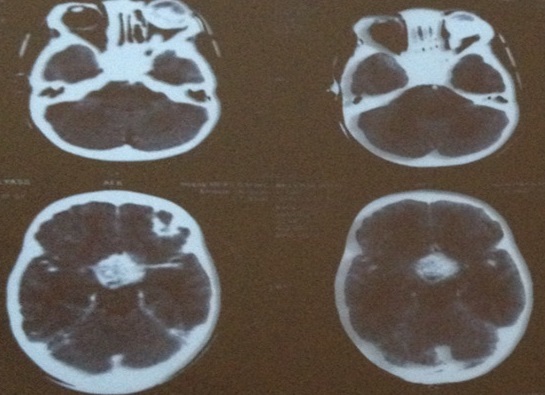
CT Scan axial cuts of a trilateral retinoblastoma with a secondary location in the pineal gland

During the study period a total of 35 eyes (26 patient) were diagnosed with retinoblastoma in our center, 12 patient (16eye) had an extra-ocular extension (46%). There were 60% males; none of our patients had positive family history. The disease was unilateral in 6 cases and bilateral in 5 patients, with one case of trilateral retinoblastoma. The average age of diagnosis was 27 months (range: 8months to 4years), however the age at presentation was higher in unilateral cases than bilateral. The initial mode of presentation was varied, there was proptosis (41, 6%) staphyloma (25%) orbital cellulitis (25%) (Figure 1) and hyphema(8,3%). However about 75% of parents had noticed leucocoria (40%) (Figure 2) or strabismus (20%) at the time before consultation, with a lag period from detection of the first sign to consultation ranging from 2 to 18 months (median of 10 months) (Table 1). The delay in diagnosis did not vary significantly between the subgroup with in unilateral and bilateral retinoblastoma. The various reasons given by parents for delayed presentation was unawareness about the seriousness of the disease (75% ), fear of enucleation (30%) limited access to specialized center and lack of appropriate finances to arrange for their travel (50.2%). Results of investigations made for the confirmation of diagnosis and for staging are listed (Table 2), and revealed involvement of orbital part of optic nerve in 58.3% (Figure 3), extrascleral involvement in 41.6%, involvement of orbital muscles in 41.6%, involvement of eyelids in 8.3%, and involvement of central nervous system in 8.3% eyes (Figure 4). The histopathology confirmed the diagnosis of the orbital involvement, scleral infiltration, choroidal and post laminar invasion. At diagnosis 11 patients had stage D of the classification ABC and one patient had stage E. All 16 eyes were managed with surgery including enucleation(75%) and exenteration (25%). Primary enucleations were performed in 33.3% of cases and 66.7% after chemo reduction; all patients received chemotherapy after surgery. One case was treated with external beam radiation for orbital recurrence. No patient developed further metastasis during a mean 30 month follow up period, orbital recurrence was observed in 2 eyes, there was one death in our study, due to neurological complications in the case presenting a trilateral retinoblastoma.

**Table 1:** Lag period between first symptom and diagnosis of extra-ocular retinoblastoma (months)

Parameters	Lag period	Range	P-value
**Lag period**			**Not significant**
Lag period according to laterality	10+/-2	2-18	
Unilateral	9	2-16	
Bilateral	12	6-18	
**Lag period according to age**			**Not significant**
0-2 years	10.5	3-18	
2-4 years	8	2-14	
More than 4 years	6.5	3-10	

**Table 2:** Imaging features of 12 patients with extra-ocular retinoblastoma

Imaging features	n (%)
Isolated extrascleral involvement	2 (16.6)
Isolated involvement of orbital part of optic nerve	1 (8.3)
Involvement of both 1 and 2	3 (25)
Involvement of the orbital muscles and optic nerve	2 (16.6)
Isolated involvement of the orbital muscles	2 (16.6)
Involvement of the orbital muscles optic nerve and eyelids	1 (8.3)
Involvement of the central nervous system	1 (8.3)

## Discussion

In developed countries, retinoblastoma is usually diagnosed in its early intraocular stages leading to high chances for preservation of vision, globe and disease free survival of the patient. However, in developing countries, retinoblastoma is often diagnosed at a later stage with extraocular dissemination, thus leading to much lower rates of ocular salvage and patient survival [11–13]. We prospectively studied 12 consecutive children (16 eyes) diagnosed with extra-ocular retinoblastoma presenting 46% of all retinoblastoma diagnosed in our center. A comparatively larger percentage of orbital extensions (52%) have been reported in a Malaysian study [14], 37.1% in India [13], 52.3% in Senegal [15]. Orbital retinoblastoma was reported in very low frequency from some of the developed countries like USA (5%) [16]. Retinoblastoma has no sex predilection; there was not much difference in the occurrence of retinoblastoma in boys (60%) and girls (40%). The average age at diagnosis is 18 months and vast majority become clinically apparent before the age of 3 years, The age at diagnosis of retinoblastoma in developing countries varies from 24 months to 3 years [13, 15]. In this study, the age of our patients ranged from 8 months to 4 years and averaged 27 months which is slightly higher. Patients with bilateral tumors present earlier than those with unilateral involvement [15]. In our series, Bilateral retinoblastoma was seen in 41,6% of patients which is similar to the figures reported from USA (41.5% ) [16], India (37.2% ) [13]. However, it was observed in very less percentage in countries like Nepal (9.3%) [9]. The mean lag period in our patients was 10 months, with no statistically significant difference as per laterality and age. Other studies from the developing world have also described a long lag period and have analyzed various reasons for the same [13, 15]. Erwenne and Franco [12] concluded that delayed presentation with extra-ocular invasion is strongly associated with age at diagnosis and lateness of referral. Antoneli [17] analyzed the lag time and concluded that longer lag time is associated with disseminated disease and this period, if shortened may lead to decrease in number of cases with extra-ocular dissemination. Another study concluded that it is not only longer lag time but also denial of management by parents that are responsible for the delayed diagnosis [13]. It is likely that parents with a poorer educational background have limited information regarding signs and symptoms of retinoblastoma. Parents often stated that even though they noted an abnormality in the eye, often leukocoria, they did not relate this to the possibility of having cancer. The various reasons reported by parents for the consultation delay in our study are limited access to ophthalmologist, lack of finances and health insurance and denial of enucleation. Another explanation rises as been reported from the use of self treatments or traditional healers' treatments before reaching a hospital [13].

Leucocoria is the most common presenting sign of retinoblastoma followed by strabismus all over the world (Table 3). The frequency of common modes of presentation of retinoblastoma in our study is consistent with many studies from different parts of the world. Leucocoria as presenting sign was seen in 22.6% [13] to 97.9%[16] of patients of retinoblastoma, while strabismus was noted in 5.6% [13] to 26% [15] of these patients at the time of diagnosis. In addition to the above, Abramson [16] reported many uncommon/rare presenting signs like anisocoria, heterochromia iridis, inflammatory signs, nystagmus, microphthalmia/buphthalmos, proptosis, orbital cellulitis, hyphema, ptosis, aniridia, phthisis, vitreous haemorrhage in their study of 1265 patients of retinoblastoma. However, proptosis was present in 41.6% of cases in our study; it was reported in high frequency from some of the developing countries like India (25.3%) [13]. Proptosis as presenting sign was reported in very low frequency from some of the developed countries like USA (0.5%) [16]. Orbital extension of the tumor results in proptosis, orbital cellulitis, lid swelling and echymosis; and the disease is considered to be in moderately advanced stage [9]. In our series, three children presented with signs of orbital cellulitis. Two children had unilateral retinoblastoma while the third child had bilateral retinoblastoma. The diagnosis was confirmed by CT scan of orbits and brain which showed tumor mass in the globe with areas of calcification in the affected eyes. Although extremely rare, orbital cellulitis as presenting sign in patients of retinoblastoma has been well documented in the literature [18]. It is suggested that necrotic changes occurring in the ciliary body and iris root trigger an inflammatory response in adjacent orbital soft tissue. Another possible route for necrotic tumor to reach out of the eye is through trabecular mesh work [18]. Histological optic nerve infiltration was noted in 75% of patients in our study which is higher than the study reported from India (32.3%) [13]. In our study we describe one case of trilateral retinoblastoma with orbital invasion and a pineal tumor, which died of intracranial complications three months after the diagnosis. Patients with germline mutations in RB1 have a risk of about 5% of developing intracranial midline primitive neuroectodermal tumors. Such a tumor in a child who typically has unilateral or bilateral familial or sporadic hereditary intraocular retinoblastoma is known as trilateral retinoblastoma [19]. With rare exceptions, trilateral retinoblastoma is located in the pineal gland or the suprasellar and parasellar region, and histopathologically, they resemble retinoblastoma. A meta-analysis [20] published in 1999, showed that 88% of children with trilateral retinoblastoma did not survive for longer than 5 years. Survival of patients with pineal and non-pineal trilateral retinoblastoma has increased. Early detection of smaller tumors predicted better survival for patients with pineal trilateral retinoblastoma. In most cases, retinoblastoma is a clinical diagnosis and is based on clinical examination and use of ancillary tests, The diagnosis of high-risk retinoblastoma is a clinical diagnosis only in cases with anterior chamber pseudohypopyon, clinically apparent iris infiltration, and choroidal or optic nerve invasion detected by imaging modalities. However, in most cases, high-risk retinoblastoma is a histopathology diagnosis that can be detected on examination of enucleated specimens [18]. There are very few human malignancies where definitive treatment is started without any confirmed histopathological diagnosis and imaging plays an important role in diagnosis and staging of the disease. Imaging (preferably magnetic resonance imaging) is required to confirm the diagnosis, access for local spread into the orbit through the sclera or into the optic nerve, metastasis into the central nervous system and for trilateral retinoblastoma [18]. Enucleation is a definitive treatment for retinoblastoma and is associated with a low complication rate but the patient can not have the choice of vision in the affected eye. with primary enucleation. Chemoreduction utilizes neoadjunctive chemotherapy to reduce the tumor volume and enable focal therapy [21].There are no proven definitive therapy or management protocols for orbital retinoblastoma. They continue to remain a challenging disease to treat because of its complex nature and usually various combination therapies are needed to achieve reasonable results. a treatment protocol comprising of initial triple drug high-dose chemotherapy (3-6 cycles) followed by appropriate surgery, orbital radiotherapy and an additional 12 cycle standard dose chemotherapy have been suggested.

**Table 3:** Comparative frequency of common presenting signs of retinoblastoma in different parts of world

Author	Year	Country	No.of patients	Leucocoria (%)	Strabismus (%)	Proptosis (%)
Badhu et al [9]	2005	Nepal	43	32.5	-	44.2
Sumitha et al [13]	2005	India	355	74.6	6.2	25.3
Sow et al [15]	2014	Senegal	53	22.6	5.6	52.8
Abramson et al [16]	1998	USA	1265	56.1	23.6	0.5
Present study	2015	Morocco Marrakech	12	40	20	41.6

## Conclusion

We do acknowledge the limitations of this study. The small numbers analyzed makes it difficult comparing our results with those from centers with larger series; similarly, it is impossible to make inferences for the whole population from this series. However the importance of the disease, being both sight and life threatening, renders the findings significant. We confirmed that intra orbital invasion is strongly associated with a delayed diagnosis due to various reasons. Many methods have been suggested for education of the public thus leading to early detection and a better prognosis for vision and life. Use of media campaigns to increase public awareness, incorporation of education program linked with national vaccination campaign, appropriate counseling of parents as not to abandon treatment and education programs for pediatricians and general ophthalmologists have all been suggested to avoid delays in diagnosis and management.

### What is known about this topic

- In developing countries, retinoblastoma is often diagnosed at a later stage with extra-ocular dissemination;
- Longer lag time and denial of management by parents are responsible for the delayed diagnosis.

### What this study adds

- Intra orbital invasion is strongly associated with a delayed diagnosis due to the unawareness about the seriousness of the disease, fear of enucleation and limited access to specialized center;
- Education of the public leads to early detection and a better prognosis for vision and life.
